# Investigation of stillbirths, perinatal mortality and weakness in beef calves with low-selenium whole blood concentrations

**DOI:** 10.4102/jsava.v87i1.1336

**Published:** 2016-07-15

**Authors:** Anthony J. Davis, Jan G. Myburgh

**Affiliations:** 1 Sterkspruit Veterinary Clinic, Lydenburg, South Africa; 2 Department of Paraclinical studies, University of Pretoria, South Africa

## Abstract

In this on-farm investigation, we report on stillbirths, weakness and perinatal mortality seen in calves on a commercial beef farm in the Roossenekal area, Mpumalanga province, South Africa. Post-mortem examination of these calves and histopathological examination of organ and tissue samples did not indicate an infectious aetiology. Affected calves had marginal to deficient whole blood selenium concentrations. Whole blood samples collected from adult cattle on this farm and five neighbouring farms were deficient in selenium. The potential contributions of other minerals to the symptoms seen are a subject of ongoing investigation, but selenium deficiency was marked in this herd and required urgent correction. Methods to correct the deficiency included the use of injectable products, and an oral selenium supplement chelated to methionine. Selenium availability to plants is primarily determined by the selenium content of the parent bedrock, the presence of other minerals and the pH of the soil. The apparent sudden onset of this problem implicates a soil factor as being responsible for reducing selenium’s bioavailability in this area. Selenium deficiency can have a significant impact on human health. HIV and/or AIDS, various forms of cancer and several specific clinical syndromes are associated with selenium deficiency in humans, and the impact on human health in this area also requires further investigation.

## Introduction

Stillbirths, perinatal weakness and mortality in calves characterised the onset of the 2014 calving season at a beef farm near Roossenekal, Mpumalanga province. Stillbirths were approaching 30% up to the point of intervention. Calves were either born dead or died shortly after birth. Severely affected calves were anaemic, moderately dehydrated, unable to suckle and unable to stand. Other calves were born normally but weakened gradually. The farmer noticed a gradual alleviation of clinical signs in weak calves after treatment with Kyrovite^®^VitE/selenium (Se) (Kyron Laboratories, Johannesburg). One week after treatment of the remaining pregnant cows with a composite mineral preparation, Multimin+Se^®^ (Virbac RSA [Pty] Ltd, Centurion), the farmer noted that no further cases of weakness or mortality were seen in calves born to these cows. Gross post-mortem findings were unremarkable, except for mucous membrane and muscle pallor. Whole blood and liver samples were submitted for laboratory Se analysis and organs sent for histopathology. In the absence of any confirmatory laboratory data, a presumptive diagnosis of selenium deficiency was made based on the clinical presentation.

Many descriptions of perinatal weakness and mortality in calves associated with selenium deficiency have been reported from other countries (Abutarbush & Radostits 2003; Cawley & Bradley 1979; Koller & Exon 1986; Lohr & Van der Wouden 1971; Waldner & Blakely 2014). An extensive review of literature revealed no description of this syndrome in calves in South Africa.

While awaiting laboratory results for selenium whole blood concentrations, other causes of perinatal mortality were considered. Bovine virus diarrhoea (BVD), chlamydophila, *Neospora caninum*, leptospirosis and infectious bovine rhinotracheitis (IBR), important infectious causes of abortion and perinatal mortality were not detected. Redwater (*Babesia bovis* and *Babesia bigemina*) is common in this area, but no evidence of these parasites was found on brain or blood smears. Iodine deficiency and mycotoxin exposure were considered, but typical post-mortal changes were absent.

The best-known manifestation of selenium deficiency in livestock is white muscle disease of lambs and calves. Lesions in cardiac muscle are usually responsible for acute death, but skeletal muscle is also affected (Orr & Blakeley 1997; Underwood 1977). Conditions associated with low-selenium blood concentration that improve when selenium is supplemented include perinatal weakness of calves, calf scours, pneumonia, poor weight gain, abortion, mastitis, metritis, retained placenta and poor fertility (Fordyce 2005; Jukola et al. 1996; Smart, Gudmundson & Christensen 1981; Underwood 1977). Some authors have defined a distinct entity characterised by perinatal weakness in calves and have termed this congenital selenium deficiency (Abutarbush & Radostits 2003; Cawley & Bradley 1979; Lohr & Van der Wouden 1971). Selenium’s importance to human and animal health is well described (Fordyce 2005; Koller & Exon 1986).

In this farm investigation, we report on clinical signs and post-mortem findings seen in neonatal calves. Whole blood selenium concentrations from cattle on five neighbouring farms were measured, and responses to various forms of supplementation were assessed.

### Materials and methods

Post-mortem examinations were performed on four calves and one lamb. Blood from the first calf was collected into EDTA vacutainer tubes for selenium determination at the University of Pretoria’s Nutrilab. Subsequent whole blood and liver selenium determination was done at Elsenburg Laboratories, Western Cape Provincial Veterinary Laboratory. Blood was also submitted to IDEXX Laboratories for creatinine kinase (CK) concentrations. Blood collected into an EDTA tube was drawn into a microhaematocrit tube and centrifuged in a microhaematocrit machine for 5 min. Urine was collected by cystocentesis, and a urine strip test (Medi-test Combi, Germany) was performed. A peripheral blood smear was stained with Rapi-Diff stain set and examined for the presence of blood parasites. A brain smear was also made, stained with Rapi-Diff stain set and examined for the presence of parasites. Thyroid glands were collected from the first two calves and weighed on an electronic scale. Organ samples from three calves were collected in 10% formalin and sent to Vetpath, IDEXX Laboratories for histopathological examination. Bovine virus diarrhoea (BVD) immunohistochemistry was performed on organ samples from the first calf. IBR immunohistochemistry was performed on the second calf. Organ samples from the fourth stillborn calf were collected, frozen and submitted to the ARC – Onderstepoort Veterinary Institute (OVI) for chlamydophila and leptospirosis polymerase chain reaction (PCR) testing.

Blood samples (*n* = 63) were collected from the caudal tail vein of adult cattle into EDTA vacutainer tubes. Blood was kept chilled and then sent on ice to Elsenburg Laboratories, Western Cape Provincial Veterinary Laboratory, for selenium determination. The method used was described by Koh and Benson (1983). Whole blood samples (*n* = 63) were digested in an acid mix, reagents were added and selenium concentration determined using a fluorimeter.

Weak calves (*n* = 13) were treated by intramuscular injection of 8 mL of Kyrovite^®^VitE/Se (Kyron Laboratories, Benrose, South Africa) injection. Adult cows (*n* = 34) were injected subcutaneously with 5 mL of Multimin+Se^®^ (Virbac). Heifers (*n* = 9) were injected with 3.5 mL subcutaneously. For oral supplementation, Sel-Plex^®^ (Alltech, Stellenbosch, South Africa), an organic (yeast-based) form of selenium, was added to the ration at the maximum allowable concentration of 0.3 mg Se per kg DMI. Calculations were based on the following assumptions: average body weight equals 500 kg and average dry matter intake equals 2.5% of body mass (dry matter intake will therefore be 12.5 kg per day). Average lick intake was estimated to be 150 g per day. Sel-Plex^®^ with a selenium concentration of 2000 ppm selenium was mixed into the lick at a rate of 12.5 kg per ton (25 ppm).

## Results

### Clinical and post-mortem findings in calves

#### Calf 1

This calf weighed 25 kg at birth, could not suckle and also could not stand. She died shortly after birth and was presented for post-mortem examination. Notable abnormalities were that the eyes were sunken and the ocular mucous membranes were paler than normal. Haematocrit was 36%. Serum was mildly icteric. Urinalysis revealed no abnormalities, except for an increased protein patch reading of 100 mg/dL. At post-mortem examination, neither ascites nor anasarca was present. Muscles were paler than would be expected in a new-born calf, and the blood took longer to clot than expected. No significant pathology could be detected macroscopically and organ samples were taken in formalin for histopathological examination. Spleen, lungs, kidney, thyroid gland, heart, brain, thymus, liver, skeletal muscle and gut were examined, but no abnormalities were detected. Thyroid glands weighed 5 g and the thyroid gland to body mass ratio was 0.0002 (normal < 0.033 [Anderson, Dalir-Naghadeh & Parkinson 2007]). The histopathologist concluded that these organ samples revealed no evidence of an infectious disease process. Blood was also collected for selenium analysis. The selenium blood concentration was 53 ng/mL. Values of 50 ng/mL or less are classed as deficient (Puls 1994). BVD immunohistochemistry was negative. CK concentrations were elevated to 932 u/L (reference range: 35 u/L – 280 u/L, IDEXX Laboratories).

#### Calf 2

Three days later, a second calf was presented for examination. The one-week-old calf was weak and could not suckle. The calf was in good condition but looked dazed and could not stand when lifted. Blood smear was negative for parasites, but neutrophilia and monocytosis were present. The owner had already treated the calf with oxytetracycline, diminazene aceturate and ketoprofen, but there was no improvement in the calf’s condition. The calf was dosed with an electrolyte solution and injected with Multimin+Se^®^ (Virbac) and dosed with oral vitamin E. Three days later, the calf was standing and able to suckle by itself. The calf continued to do well but, 23 days later, had a relapse and was unable to stand. The calf was then injected with VitE/Se^®^ (Kyron) and then began to move its legs 6 h later. The calf improved but died on the farm 4 days later. Whole blood selenium measured 235 ng/mL and liver selenium 4.35 mg/kg (normal: 0.5 mg/kg – 3 mg/kg) at time of death (27 days after Multimin+Se^®^ [Virbac] injection and 3 days after VitE/Se^®^ [Kyron]) injection. On post-mortem examination, a putrefactive rumenitis with fetid rotting milk was found macroscopically. No additional organ pathology to indicate the presence of a primary infectious disease process was noted on histopathological examination. It was noted, however, that the thymus was significantly atrophic.

#### Calf 3

This calf was weak at birth, pale, unable to stand and could not suckle. She died 4 days after birth and weighed 22 kg at the time of death. No VitE/Se was given to this calf. Blood selenium concentration was 37 ng/mL, which is classified as deficient and liver selenium was 0.83 mg/kg (normal: 0.5 mg/kg – 3 mg/kg), which is classed as marginal (Table 1) (Puls 1994). Blood smear was positive for *Anaplasma marginale* and negative for *B. bovis* and *B. bigemina*. A brain smear was negative for *B. bovis* and *Ehrlichia ruminantum*. A post-mortem examination was performed. The carcass was mildly icteric. Cranial lung lobes were dark red and consolidated. The thyroid gland was asymmetrical and weighed 7 g. The thyroid gland to body mass ratio was 0.0003% (normal < 0.033 [Anderson et al. 2007]). On histopathology, haemorrhagic pneumonia with bacteraemia was detected, but no indications of a primary infectious cause could be found. Immunohistochemical staining for IBR was negative. No histopathological lesions indicative of *N. caninum* or *Toxoplasma gondii* infection in brain or spinal cord specimens could be seen. No thyroid gland pathology was detected.

**TABLE 1:** Classification of selenium status in liver and whole blood.

Selenium status classification	Liver mg/kg DM†	Whole blood ng/mL‡
Deficient	< 0.2	< 50
Marginal	0.2–0.5	50–80
Adequate	0.5–3	80–1200
High	> 6	> 3000

*Source*: Adapted from Van Ryssen, 2001

† Caple and McDonald (1983)

‡ Puls (1994).

#### Calf 4

Organ samples from a fourth calf submitted to the ARC-OVI tested negative for chlamydophila and leptospirosis on PCR examination.

#### Lamb

Two months later, on the same farm, a four-month-old lamb died acutely and was presented for post-mortem examination. Organs samples were sent to Vetpath, IDEXX Laboratories for histopathological examination. Chronic myocardial necrosis, fibrosis and calcification were observed histologically. These lesions characterise a vitamin E/Se deficiency. Selenium blood concentrations were 88 ng/mL (normal range: 100 ng/mL – 500 ng/mL) and liver selenium was 0.82 ng/mL (normal range: 0.5 ng/mL – 3.00 ng/mL). These selenium concentrations are classified as marginal by Puls (1994) (Table 1).

### Whole blood selenium concentrations

Multimin+Se^®^ (Virbac) was administered to 34 cattle on the farm (Herd 1) in which the stillbirths occurred. Average whole blood concentrations measured 6 weeks after injection were 94.1 ng/mL. Whole blood selenium concentrations of 7 out of the 34 cattle were < 80 ng/mL, classified as marginal, and 27 of these cattle measured > 80 ng/mL, classified as adequate. Unsupplemented cattle from the same farm (Herd 1) were clearly deficient in selenium (Table 2, Herd 1). Selenium whole blood samples collected from 9 cows, 30 km from Herd 1, were also low (Table 2, Herd 2). In addition, cattle from 4 other neighbouring farms (Herds 3–6) were bled and average whole blood selenium concentrations were in the deficient range (Table 2).

**TABLE 2:** Pre-supplementation whole blood selenium values of cattle from 6 different farms within 50 km radius of Herd 1.

Herd number	Average selenium whole blood levels (ng/mL)	s.d.	*n*
HERD 1	24.0	4.8	9
HERD 2	25.4	11.1	9
HERD 3	27.0	11.7	19
HERD 4	47.3	14.3	9
HERD 5	40.3	8.3	6
HERD 6	45.5	16.6	11

**Average**	**34.9**	**11.1**	**-**

*n* = 63.

Based on low whole blood selenium concentrations and compelling clinical evidence, a short-term supplementation programme was instituted. Two different injectable selenium and vitamin E/Se-containing products were used and a 1.8–2.9 times increase in selenium whole blood concentrations was measured in 14-month old heifers after injection with Kyrovite Vit E/Se^®^ (Kyron) and Multimin+Se^®^ (Virbac), respectively (Table 3). Whole blood selenium concentrations measured 6 weeks after oral supplementation with Sel-Plex^®^ (Alltech) had also increased. However, since these cattle were given an injectable formulation 3 weeks prior to this, it is not possible to determine to what extent the oral supplementation contributed to this increase and what contribution the injectable mineral formulations made to these blood concentrations. Blood concentrations measured 6 months after commencement of the supplementation programme were 4.8 times higher than pre-supplementation (Table 4).

**TABLE 3:** Dose–response trial to monitor the response of individual cattle (14-month-old heifers) to two injectable formulations and an oral supplement: Whole blood selenium concentrations (ng/mL) were measured 3 weeks after an injection with either 8 mL of VitE/Se^®^ (Kyron) or 3.5 mL of Multimin+Se^®^ (Virbac) and 6 weeks after oral supplementation with Sel-Plex^®^ (Alltech).

Heifer ID	Initial values (Whole blood Se ng/mL)	Post-Multimin (Se ng/mL)	Increase†	Post-VitEse (Se ng/mL)	Increase†	Post-Sel-Plex (Se ng/mL)	Increase†
13–53	21	64	3	-	-	-	-
13–20	26	84	3.2	-	-	-	-
13–70	20	56	2.8	-	-	-	-
13–54	22	79	3.6	-	-	134	1.7
13–21	35	78	2.2	-	-	134	1.7
13–58	26	63	2.4	-	-	132	2.1
13–66	19	-	-	40	2.1	83	2.1
13–18	25	-	-	35	1.4	77	2.2
13–55	22	-	-	40	1.8	71	1.8

**Average**	**24**	**70.7**	**2.9**	**38.3**	**1.8**	**105.2**	**1.9**

† Increase is calculated by dividing the whole blood selenium concentration value prior to treatment by whole blood selenium concentration after treatment.

**TABLE 4:** Average increase in whole blood selenium concentrations of randomly bled cattle from Herd 1 and Herd 2, 6 months after supplementation programme was introduced.

Herd data parameters	Before supplementation (November): Se level (ng/mL)	After supplementation (May): Se level (ng/mL)
Average whole blood selenium levels	35.8	170.6
*N*	20	16
Standard deviation	16.6	48.5
Proportion of animals with deficient selenium whole blood concentrations	17/20	0/16

## Discussion

No histopathological lesions to indicate an infectious or parasitic process were detected in any of the post-mortem examinations done on the first three calves, including immunohistochemical stains for BVD in calf 1 and IBR in calf 3. Iodine deficiency causes perinatal weakness in calves, but thyroid gland ratios were normal and pathology associated with goitre was not present. Putrefactive rumenitis due to failure of abomasal groove closure is a common complication in tube-fed calves. The poor suckling reflex results in a reduced colostral intake that impedes the passive transfer of antibodies, so these calves are commonly immunocompromised. Secondary infections are therefore to be expected: *A. marginale* was seen on blood smear and haemorrhagic pneumonia was diagnosed in calf 3. Thymic atrophy noted in calf 2 could be associated with selenium deficiency (Waldner & Blakely 2014).

The pathology in muscle and myocardial tissue found in the lamb are typical and confirmatory of a vitamin E and selenium deficiency. Tustin (1959) reported white muscle disease in South African sheep.

The fact that blood samples collected from unsupplemented adult cattle in Herd 1 and from neighbouring farms (Herds 2–6) were selenium-deficient, confirms that this problem is not unique to this farm but affects a wider geographical area (Table 2). Injectable products can raise blood concentrations rapidly in the short term (Table 3). However, since Multimin+Se^®^ (Virbac) is a relatively short-acting product (up to 3 months), a longer term solution by means of oral supplementation was required.

Oral supplementation effectively increased blood selenium concentrations (Tables 3 and 4). Organic oral selenium supplements are chelated to methionine and more readily absorbed than selenite (Ammerman et al. 1980; Gunter, Beck & Philips 2003). They are also reported to be more effectively transferred transplacentally (Kincaid 1999). It has been demonstrated that if dams are supplemented from 125 days of gestation with this form of selenium supplement, their calves are born with high selenium blood concentrations and they maintain these blood concentrations until weaning (Davis et al. 2005). Whole blood selenium concentrations were measured 6 weeks after oral supplementation with Sel-Plex (Alltech) commenced and these concentrations increased (Table 4).

Measurement of selenium whole blood concentrations is an indirect measure of the clinical benefits of selenium supplementation. It is impossible to measure selenium concentrations in all enzyme systems in which selenium exerts an influence. Cytosolic glutathione peroxidase is only one of many enzyme systems into which selenium is incorporated (Birringer, Pilawa & Flohe 2002). Nevertheless, the important change from a clinical point of view is that an increase in selenium whole blood concentration means that more selenium is available but does not give an accurate indication of how well this selenium has been incorporated into the enzyme systems in which it is required. In a review of assessment of trace mineral status of ruminants, Kincaid (1999) stated that ‘perhaps the ultimate assessment tool is the response of animals to supplementation’. Blood concentrations are a guideline, but an improvement in production and a reduction in the number of weak and stillborn calves will be the only true indication of whether supplementation was successful or not.

Although the onset of the stillbirths and weak calves was a sudden event, in retrospect, changes associated with selenium deficiency were present for some time before this. Over the past 2 years (2013–2014), the farmer noticed a drop in conception rate and poor weight gain in the calves. The herd tested negative for brucellosis in June 2014, but the farmer did note an increase in unexplained abortions. These subtle signs do not readily alert to the possibility of a selenium deficiency, as they are non-specific but have been associated with selenium deficiency by various authors (Andrews 2003; Koller & Exon 1986; Underwood 1977). The farmer also recalled problems during the previous year’s calving season: the calves were small, but they were not expelled easily. The birth weights of calf 1 and calf 3 are low, but they were both full-term calves. They were born alive, were hirsute and well-developed.

An important observation is that selenium deficiency alone is not necessarily associated with clinical signs (Koller & Exon 1986). The condition is multifactorial. Muscle exertion is a trigger for the onset of symptoms in older calves. This has been described in yearling cattle put out to pasture (Andrews 2003). While confined no signs were seen, but the increased muscle activity after release resulted in clinical myopathy. Considering the manifestation of signs seen in these calves, muscular exertion during the calving process could have precipitated them. Ensuring that selenium concentrations are within the normal range is likely to prevent the manifestation of clinical signs associated with muscle exertion. Treatment with vitamin E will supplement the anti-oxidant role played by selenium but cannot replace it (Bus, Aust & Gibson 1976).

Selenium taken up into mammalian systems follows distinct metabolic pathways until its final inclusion into glutathione peroxidise or other selenoproteins. Selenium may be taken up in different forms but is eventually metabolised to selenide, from which either selenoproteins are formed, or, after combining with cysteine, selenocysteine, the precursor molecule to glutathione peroxidase. A pathway also exists for the excretion of excess selenium (Birringer et al. 2002).

The biological significance of selenium was first appreciated after the discovery that selenium is an essential component of the important anti-oxidant enzyme, glutathione peroxidase (Rotruck et al. 1976). In addition to the four known groups of glutathione peroxidase, at least 14 selenoproteins are responsible for other anti-oxidant roles (Birringer et al. 2002). An interesting phenomenon in selenium biochemistry, which will influence recovery following selenium supplementation, is that incorporation into different enzyme systems follows a specific pattern, based on the availability of selenium. An extensive review of selenium biochemistry has been compiled by Birringer et al. (2002).

A deeper concern is the impact that this selenium deficiency may have on the local rural human population. If the soil in this area is severely selenium deficient, people living here who do not have access to food produced elsewhere will most likely be selenium-deficient. This is a form of ‘geological entrapment’, a core concern of medical geology (Davies et al. 2005). It is well described that HIV patients with inadequate selenium intake progress more rapidly to clinical manifestations of the disease (Birringer et al. 2002; Melse-Boonstra, Hogenkamp & Lungu 2007). Several clinical syndromes associated with selenium deficiency have been described in humans, namely Keshan disease, Kashin-Beck disease, iodine deficiency and cardiovascular disease (Fordyce 2005). Selenium deficiency is also associated with the progression of various different types of cancer, including colorectal, prostate, skin and liver cancer (Fordyce 2005; Yu et al. 1991).

The primary determinant of selenium content in soil is the geological composition of an area (Alloway 2005). However, the interactions involved in the uptake of selenium in plants and bioavailability of selenium to plants are affected by a number of factors. Soil pH plays a pivotal role in the availability of selenium, as this alters the ionised state of selenium. Selenate (Se^6-^), a more soluble, easily bioavailable ionic form of selenium is more prevalent in alkaline soils, whereas selenite (Se^2-^), which is less soluble and therefore less bioavailable, predominates in acidic soils (Lindh 2005). Various cations, notably iron (Fe^2+^ form), if present in the soil, can also potentially reduce the availability of selenium to plants. Different plant types accumulate selenium at different rates, so this is another important variable to consider.

Van Ryssen (2001) reviewed the selenium content of South African soils, but during personal discussions in November 2014, he stated that he has no data on selenium concentrations of soil in the Roossenekal area. The poor availability of selenium to plants is well illustrated by a study done to determine the selenium content of maize from South African silos. Despite maize grown in areas regarded as having adequate soil selenium concentrations, 96% of 896 maize samples collected from all over the country were found to be deficient in selenium (Courtman, Van Ryssen & Oelofse 2012). In these same areas, free-ranging game was also found to be selenium-deficient (Van Ryssen 2006). It was hypothesised that a soil factor, most likely a lowering of the pH, reduced the bioavailability of selenium to vegetation (Courtman et al. 2012). Soil-buffering capacity can be affected by acid rain and this could play an important role in limiting the bioavailability of selenium in affected areas (Davies & Mundalamo 2010; De Villiers & Mkwelo 2009; Van Ryssen 2001). The Roossenekal area is not far from active industrial areas and is also a site of mining activity.

## Conclusion

This preliminary study not only clearly identifies selenium as a deficient mineral in the beef herd initially investigated but also in animals from other farms in the district that were tested. Unfortunately, it is not yet possible to state with certainty that selenium deficiency alone caused the clinical signs seen in these animals. A more detailed investigation assessing the role of other minerals and vitamins, as well as additional screening for toxic and infectious agents, will be required to rule out other contributing factors to the signs seen in these calves. An urgent priority is to measure the selenium content of the soil and to investigate factors which may be responsible for a reduction in bioavailability of selenium to vegetation. Cattle on adjacent farms also have low whole blood selenium. A survey should be conducted to appreciate the geographical extent of this problem. Geologically entrapped people living in this area are also at risk of a deficiency. The impact that this deficiency has on HIV patients is well described and must be a subject of further study. Selenium may be a trace mineral, but it is essential to both humans and animals: a truly ‘One Health’ mineral. The negative impact on animal production and the potential to compromise human health have significant socio-economic consequences, which justify further investigation of the deficiency in this area and over a wider geographical area.
